# Design and Simulation Study of the Induction Heated Injection Mold with Sliders

**DOI:** 10.3390/ma14237476

**Published:** 2021-12-06

**Authors:** Paweł Muszyński, Przemysław Poszwa, Krzysztof Mrozek, Michał Zielinski, Piotr Dalewski, Michał Kowal

**Affiliations:** 1 Institute of Mechanical Engineering, Poznan University of Technology, Piotrowo 3, 61-138 Poznan, Poland; pawel.muszynski@put.poznan.pl (P.M.); dalewski.piotr95@gmail.com (P.D.); michal.kowal@put.poznan.pl (M.K.); 2 Institute of Materials Technology, Poznan University of Technology, Piotrowo 3, 61-138 Poznan, Poland; przemyslaw.poszwa@put.poznan.pl

**Keywords:** injection molding, selective induction heating, rapid temperature cycling (RTC)

## Abstract

In order to increase the quality of the products manufactured by injection molding, RTC technology can be used to achieve higher mold temperatures. As a result, the path of the injected melt can be extended, allowing the production of parts with more complex shapes and greater length. Induction heating allows heating only selected forming surfaces of the mold which increases the speed and efficiency of the process. This paper presents the concept of a detachable inductor integrated with sliders to enable the application of this technology in an injection mold with sliders, along with the theoretical model used to perform the tests. First, the effect of the magnetic concentrator shape on the process was analyzed. This was followed by a simulation study of the influence of process parameters: heating time, frequency, and electric current. An extensive analysis of the test results of the temperature distribution on the insert allowed for the selection of parameter sets that would enable obtaining the desired surface temperature without a major increase in process time. The results of simulation studies confirm the possible applications and present the range of parameters for obtaining the optimal process.

## 1. Introduction

The injection molding process makes it possible to manufacture products with good strength and aesthetic parameters, while remaining cost-effective for large-scale production [1,2,3]. It is currently the most popular method of manufacturing plastic parts [4] as a result, and the items produced this way are used in many industries [5,6]. In order to increase process speed by reducing the time needed for the material to solidify and to reduce the amount of material needed for production, thin-walled parts are manufactured. It is important in this case to maintain process repeatability and reduce the number of defective products. For conventional constant temperature molds this can be particularly difficult to achieve especially for more complex part shapes and parts with greater lengths. This is due to the difference between the temperature of the molding surface and the temperature of the injected material. As a result of this difference, the melt flowing in the cavity is gradually cooled during the injection process. This translates into an increase in viscosity with the distance traveled during cavity filling, and thus an increase in flow resistance [7]. This also causes a reduction in the flow cross-section by the formation of layers of already solidified plastic in the path. Under these conditions, it is very difficult to fill the entire mold cavity, especially when materials with increased viscosity or with the addition of fillers are used in the process [8]. Therefore, defects may appear on products such as diesel effect, short shots, warping, visible welding lines and others [9]. These issues lead to the necessity of increasing the injection pressure in conventional molds, but still the presence of defects can be unavoidable and can lead to additional flaws in the product. Both these groups of product defects can be removed by additional technological processes, but it is more beneficial to receive the finished product only by injection molding for economic and ecological factors.

As it can be concluded from the description of the injection process issues, the most important parameter is the temperature of the forming surfaces. It was also confirmed by the studies [10,11,12]. Rapid Temperature Cycling (RTC) technology can be used to solve these problems. The process involves cyclic heating of the surface of the molding insert to a temperature close to that of the flowing material during injection molding [9,13]. After the cavity is filled, the cooling phase follows. This cycle is adapted to the operation of the injection molding machine and the technological parameters. This allows to achieve a lower pressure difference in the molding cavity, which results in its better filling, especially in the case of thin-walled products [14]. This results in better quality molded parts with fewer defects [15].

The pursuit of producing better quality goods is driving the development of new heating methods for heating the molding surfaces of injection molds [16,17,18,19,20]. Induction heating technology is under investigation, also due to the possibility of heating only the desired areas instead of the entire mold like in the case of steam heating or cartridge heating. This results in a shorter mold cooling process. The advantage of induction heating is also high heating rates and small heating volume. This is especially noticeable during high frequency heating. Work on increasing the efficiency and stability of the process was carried out, among others, by Chen et al. [21,22,23,24]. Research is also underway on new solutions aimed at increasing the control of heated areas and at achieving higher heating rates [25,26,27]. The main factor verifying this technology is the obtained properties of the manufactured products. This has been discussed in the paper [28] where the authors analyzed the effect of selective heating on the production of electrical connector housing. Improved molding surface quality was achieved by using molding surface mapping while achieving heating rate of 10 °C/s [29]. The use of induction heating also results in a longer melt flow path. This is confirmed by the results presented by Lee et al. in which they analyzed the products manufactured by using polymer/metal hybrid molding technology in which a metal component, that is part of the molded part, was subjected to induction heating [30]. Other RTC methods also make it possible to achieve heating of the forming surfaces and to improve product quality. An example is the use of heating technology with assisted gas [31], with which it was possible to obtain an increase in melt flow path from 38.6 to 170 mm for a temperature of 400 °C and a heating time of 20 s. Better weld line quality was obtained by Huang et al. by using infrared heating for mold forming surfaces during long-fiber polymer molding [32].

A magnetic concentrator can be used to reduce the heating of the rest of the mold outside the forming surfaces during induction heating. It concentrates the magnetic flux lines on the area intended to be heated [33]. This material is made of magnetic powders and a dielectric binder combined in a technological process at high temperature and high pressure. It is characterized by low conductivity, low magnetic losses and high permeability [34]. The main advantages of the magnetic concentrator used to increase the intensity of induction heating is the increase of process efficiency and thus the reduction of heating by directing more heat to the desired areas, also the energy consumption is reduced. A shorter time is also achieved for heating the forming surface to the required temperature [33].

This paper presents the concept of a detachable inductor integrated with sliders for heating the forming surfaces of an injection mold with sliders. Simulation studies of mold insert heating without and for different shapes of magnetic concentrator were performed, analysis of the results were also carried out. For the selected shape of the concentrator, a range of simulation studies was then performed in which the effect of process parameters on the distribution of temperatures on the surface and in the insert was examined. The influence of the current frequency [kHz], heating time [s] and electric current [A] flowing through the coils were successively studied. Then, groups of parameters were chosen, thanks to which it would be possible to meet the requirements for the process, i.e., to obtain the forming surface temperature of 200 °C and the heating time no longer than 4 [s]. The conducted tests and analyses confirm the possibility of application in an injection mold with sliders and allow selection of the range of parameters for such a process.

## 2. Materials and Methods

### 2.1. Detachable Inductor Integrated with Sliders

**Figure 1:**
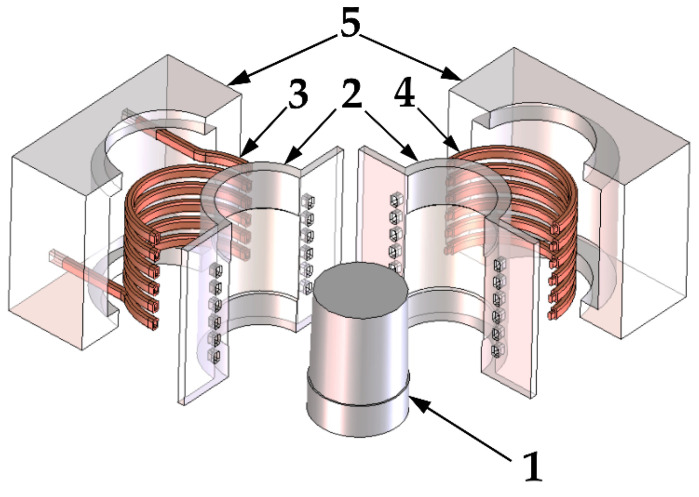
Visualization of the detachable inductor integrated with sliders, 1—molded sleeve shaped part, 2—molding inserts, 3—part of induction coil, 4—part of induction coil, 5—slider bodies.

In this paper, a detachable inductor solution for an injection mold with sliders is investigated. A mold producing sleeve shaped parts (number (1) on Figure 1) has been taken for analysis. The visualization of such an inductor is shown in Figure 1, its main components are the molding inserts (2), the inductor coil composed of two elements (3 and 4) and the sliders bodies (5). The coils (3, 4) are placed behind the inserts (2), as shown in the figure. The shape of the formed part is reflected on the surface of the inserts, and their task is to shape the injected material. Thanks to them, the desired shape of the product is achieved.

In the described concept, when the mold closes, the sliders are pushed together, and the coils of the inductor are connected. This closes the magnetic field loop around the molding surfaces. The increased efficiency of the process is achieved by designing the coil in such a way that the directions of the current flowing through the adjacent coils are consistent with each other. The presented construction of the inductor causes that the heating takes place only when the mold is closed. Only one induction generator is necessary for the system to work properly. In the slider body, behind the inductor coils, a magnetic concentrator (not shown on Figure 1) will be placed to concentrate the magnetic field on the forming insert. This will increase the efficiency of the process and increase the temperature of the insert during injection molding.

### 2.2. Theoretical Model

The process of induction heating of forming surfaces in the presented solution has been analyzed by conducting simulation studies. For this, a model of the induction heating process based on the one presented in the paper [9] by Mrozek and Chen was used. The basic input parameters for the process are the geometry of the slider system with a detachable inductor integrated with it. Along with the geometrical specifications, the material parameters of parts were included. The next process parameter is the heating time *t* [s]. Furthermore, the electric current parameters were added, i.e., the current frequency *f* [kHz] and electric current *I* [A]. After defining the input parameters, the first stage of calculations is the electromagnetic analysis. It is based on Maxwell’s equations [35] and allows to describe the distribution of electricity and energy in the material in relation to the distribution of the magnetic field. The equations employed are:

(1) $$\nabla \times E=-\frac{\partial B}{\partial t},$$

(2) $$\nabla \times H=J+\frac{\partial D}{\partial t}=J_{s}+J_{e}+\frac{\partial D}{\partial t},$$

(3) ∇·D=ρ,

(4) ∇·B=0,

where: *E*—strength of an electric field, *B*—magnetic induction, *t*—time, *H*—magnetic field intensity, *J*—current density, *D*—electric displacement, *J_s_*—source current vector, *J_e_*—induction current vector.

As a result, the values of magnetic field *B* [*T*] and magnetic field intensity *H* [A/m] for the investigated system are calculated. This allows proceeding to the next stage of calculations, i.e., transient thermal analysis. In this part, the following equations are used:

(5) $$\frac{\partial I^{e}}{\partial T_{i}}=\left[\left(K_{1}^{e}+K_{2}^{e}\right)\right]\left\{T^{e}\right\}+\left[K_{3}^{e}\right]\left\{\frac{\partial T^{e}}{\partial t}\right\}-p=0,$$

(6) $$\dot{q}=Re\left[\frac{1}{2n}\sum_{i=1}^{n}\rho J_{ti}\dot{J_{ti}}\right],$$

(7) $$\delta =\sqrt{\frac{\sigma }{\pi f\mu_{r}\mu_{0}}},$$

where: *I^e^*—current density, *T*—temperature, *K^e^*—coefficient matrices, *t*—time, $\dot{q}$—heat generation rate, *Re*—real component, *ρ*—density, *J_ti_*—current density in element, $\dot{J_{ti}}$—conjugate values of current density, *δ*—skin depth, *σ—*resistivity of the material, *f—*current frequency, *μ_r_*—relative magnetic permeability of the conductor, *μ*_0_—permeability of free space.

**Figure 2:**
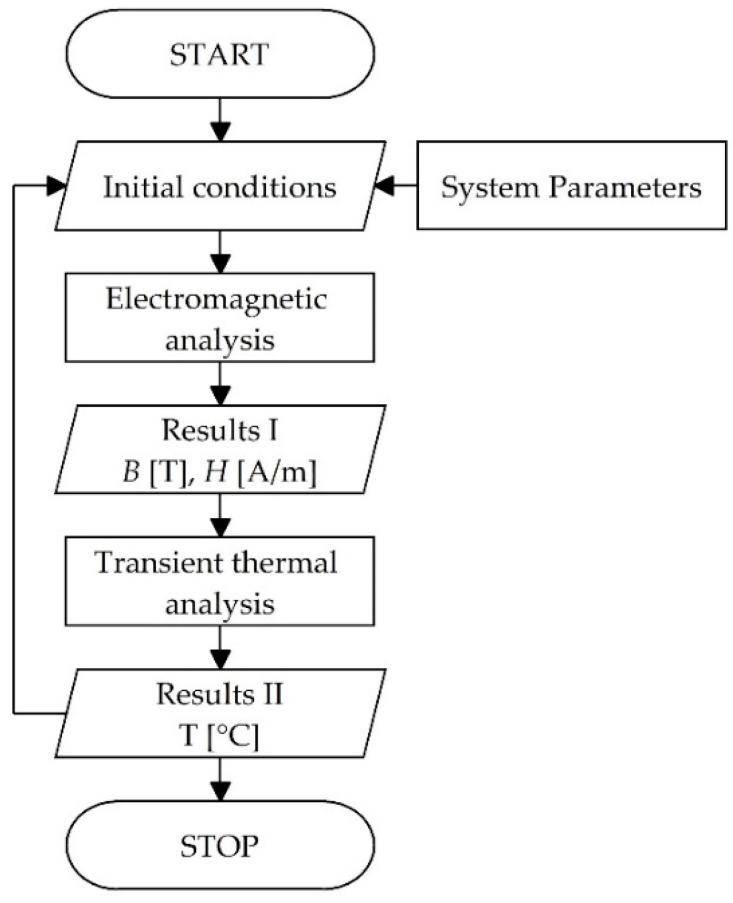
An algorithm used to perform simulation experiments.

A flowchart of the algorithm with the described model used to perform the simulation study is shown in Figure 2. As a result of such tests a description of the induction heating process of the system is obtained, consisting of the magnetic flux density distribution, magnetic field strength, generated Joule heat from eddy current losses on the heated element, as well as a non-stationary thermal analysis determining the temperature changes as a function of time.

### 2.3. Experimental Procedure

Simulation studies of the induction heating process of the slider forming insert have been carried out by using finite element method (FEM) QuickField 6.3.1 package (Tera Analysis, Svendborg, Denmark). The 2D cross-sections of the presented solution with the use of local mesh thickenings around the inductor and the slider forming insert were used.

The geometry of the coil is chosen in the model to meet the design assumptions and is unchanged throughout the series of tests performed. The dimensions of the molded part were assumed to be 50 mm in height and 20 mm in diameter, and the inductor parts were designed according to these dimensions. The cross-section of the coil is rectangular with dimensions of 4 mm by 6 mm, with an edge rounding radius of 1 mm. The longer edge is oriented along the forming insert, the pitch between the coils is 10 mm. Heated forming insert thickness is 6 mm. The material of the inserts and their bodies was hot-work tool steel 1.2343, commonly used in the production of injection mold inserts. The parameters of the materials used in the simulation are presented in Table 1.

The following assumptions were made for the process parameters. The heating time should not exceed 4 s in order to not prolong the process cycle. The temperature on the forming surface should be between 200 and 250 °C, and the maximum temperature of the forming insert near the inductor should be 400 °C in order not to damage the mechanical properties of the steel from which the insert is made. It was assumed that the surface is continuous at the point of closure of the sliders. The parameters that were analyzed during the study were those listed in the theoretical model as input parameters except for the material parameters that were constant during the simulation tests.

## 3. Results

### 3.1. Investigation of the Influence of the Magnetic Concentrator Shape on the Heating Process of the Forming Insert

**Figure 3:**
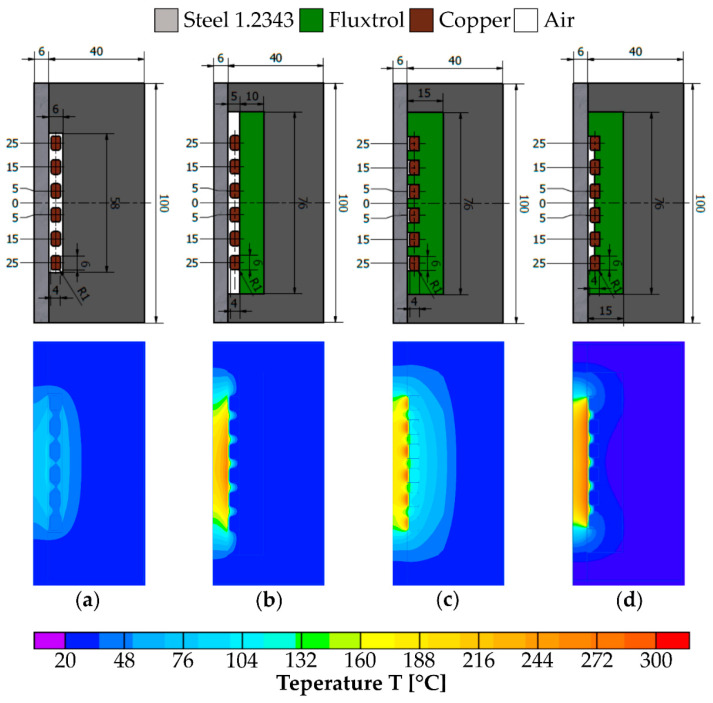
The investigated variants and results of experiments (all dimensions in millimeters [mm]): (**a**) no magnetic concentrator; (**b**) magnetic concentrator behind coil turns; (**c**) coil surrounded by a magnetic concentrator; (**d**) coil surrounded by a magnetic concentrator with height limited to half between turns.

The first stage of the research was to examine the influence of the magnetic concentrator on the heating process of the forming insert surface. The initial solution tested was a variant without magnetic concentrator in which the slider body is located behind the inductor coil. Then three possible shapes of the concentrator were examined. All four variants (a–d) are shown in the form of the slider cross-section in the upper part of Figure 3.

**Figure 4:**
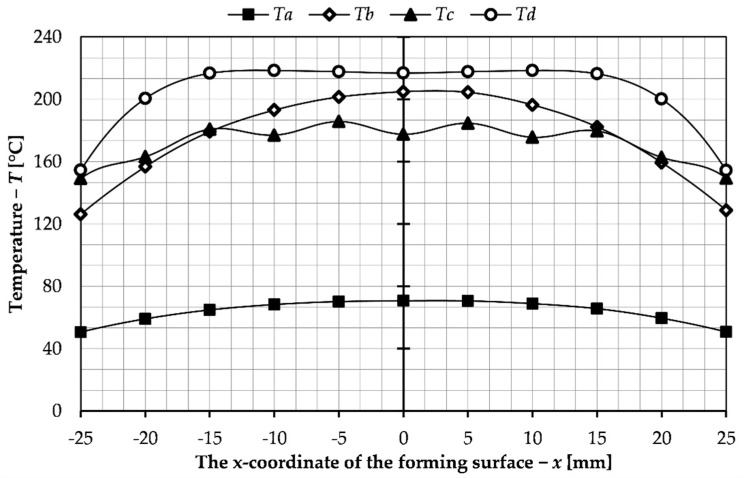
Temperature distribution on the surface of the forming insert for the variants investigated: Ti—temperature on the surface of the forming insert for the variant, i—variant symbol.

All the simulation tests were carried out for the current of 500 A, frequency of 20 kHz and heating time of 5 s. The tests results are presented in Figure 3, which shows the temperature distribution across the section of the slider where the forming insert is located. Figure 4 shows the continuation of the results that include the surface temperature of the forming insert.

The least suitable temperature distribution was obtained for variant (a), which can be seen on the results shown on Figure 3 and Figure 4. This is due to the lack of magnetic concentrator, which causes, apart from heating of the forming insert, heating of the slider body. The lowest forming surface temperature is obtained for this solution. In order to direct the magnetic flux towards the insert, a magnetic concentrator has been used in the subsequent variants. Thanks to that, as can be seen from the presented temperature distributions (variants b–d), it was possible to achieve a higher temperature of the insert forming surface and to reduce heating of the slider body. For variant (b), the concentrator was positioned behind the coil turns. This resulted in an improved temperature distribution, but the desired surface temperature was reached only at the center of the forming insert. To obtain an even temperature on the surface, the shape of the magnetic concentrator in variant (c) was changed so that it encloses the inductor on three sides. As can be seen from the results for this variant (Figure 3 and Figure 4), this improved the uniformity of heating of the forming insert surface, but there occurs heating of the slider body and formation of local zones with elevated temperature. To eliminate these undesirable phenomena, which can lead to damage of the tool, the concentrator was further modified; the height of the concentrator between turns of the coil was changed so that it is half the cross-sectional width of the coils (Figure 3d). As a result of these changes, an even temperature distribution of the forming insert was obtained and unfavorable phenomena such as heating of the slider body and occurrence of high temperature zones were eliminated. For these reasons variant d has been chosen for further simulation studies aimed at analysis of the process parameters.

### 3.2. Investigation of the Influence of Process Parameters on the Heating of the Forming Insert

**Figure 5:**
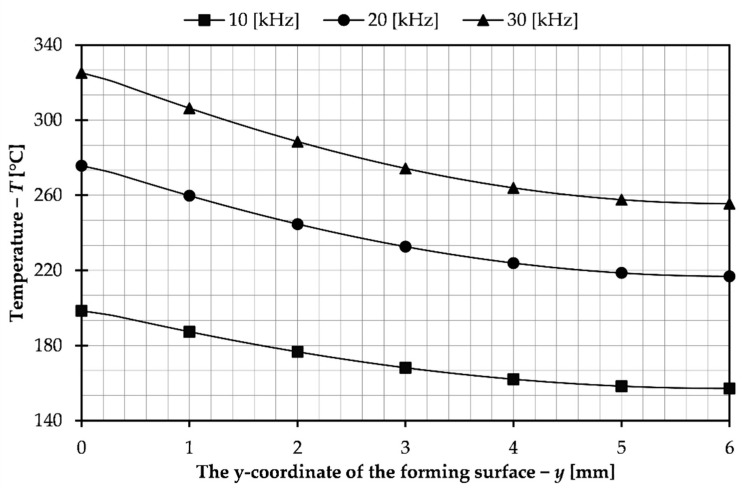
Temperature in the depth of the forming insert for different current frequencies.

In this part of the paper, a simulation study of the influence of process parameters on the heating of the forming insert was carried out. As the first step, the effect of the frequency of the electric current on the temperature of the forming insert was studied. The tests were carried out for frequencies of 10, 20 and 30 kHz and for the same electric current and heating time. The obtained results are presented in the graph in Figure 5 which shows the temperature in the depth of the insert.

As can be seen in the presented graph, the highest surface temperature was observed at the highest tested frequency of the electric current, i.e., 30 kHz. The skin effect is also relatively small, which results from the structure of the system. The forming cavity is a core placed inside the coil. The skin effect would occur in a more intense level during surface heating [27,28]. It relies on the fact that as the current frequency increases, the depth of penetration of the magnetic field lines into the material decreases. In the presented solution, the aim is to obtain a uniform surface temperature of the insert and to avoid any excessive overheating. For these reasons, based on the results obtained, a lower frequency of 10 kHz was chosen for further studies.

**Figure 6:**
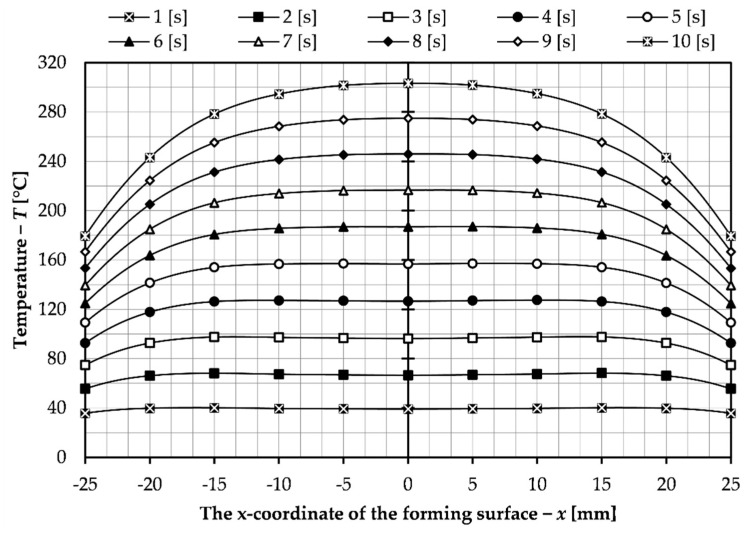
Temperature distribution on the surface of the forming insert for different heating times.

Another parameter investigated is the heating time. As stated in the materials and methods section of the article, this time should not exceed four seconds in order not to prolong the process cycle. Nevertheless, in this section heating times higher than 4 s were also investigated in order to compare a wider range of data. The simulation studies were carried out for a pre-selected variant of the magnetic concentrator shape and a current frequency of 10 kHz, and for this part of the study an electric current of 500 A was chosen. The range of heating time that was tested was from 1 to 10 s with a step of 1 s. The results obtained from the simulation tests are summarized in the graph in Figure 6 which shows the temperature distribution on the surface of the slider forming insert.

**Figure 7:**
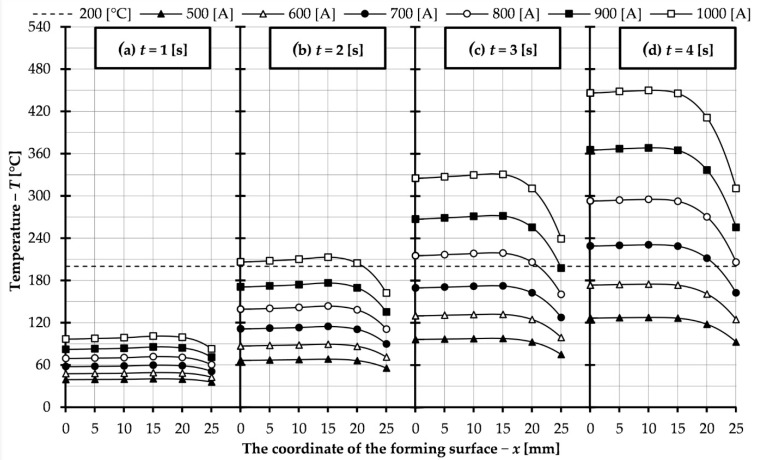
Temperature distribution on the surface of the forming insert for different electric currents flowing through the inductor: (**a**) heating time *t* = 1 s; (**b**) heating time *t* = 2 s; (**c**) heating time *t* = 3 s; (**d**) heating time *t* = 4 s.

As can be seen from the presented graph, with increasing the duration of the heating cycle, the irregularity of the surface temperature of the forming insert increases. For the parameters in this part of the study (i.e., f = 10 kHz, I = 500 A), the obtained heating time to achieve a surface temperature of 200 °C is 7 s. This is too long due to the length of the process, so in the following part of the paper, tests were performed for different values of current flowing through the inductor. The heating time range was limited from 1 to 4 s and the current range from 500 to 1000 A with a step of 100 A. The results obtained are presented in Figure 7 and show the temperature distribution on the surface of the insert. Due to the symmetric distribution, only results for half of the surface, from the center to the end of the insert, are presented. In this way it was possible to compile the results for all investigated heating times on one diagram. The temperature of 200 °C, which is the desired forming surface temperature, is also marked on the graphs. In addition, Table 2 shows the collected average, median, and standard deviation values for the temperature distribution over the entire forming surface (from −25 to 25 mm) for data comparison and analysis.

The results of simulation studies for a heating time of 1 s (Figure 7a) did not allow achieving a surface temperature of 200 °C, so it can be determined that for the given process parameters the time must be longer. As can be seen from the data shown for times 2, 3 and 4 s it was possible to obtain the desired temperature. As the heating time increased, 200 °C on the surface could be obtained for a lower electric current. Thus, the assumption that the process cycle would not be extended was achieved.

The results presented in Table 2 allow to observe the effect of process parameters on the temperature distribution on the forming surface of the slider. This can be seen in the difference between the average and median value and in the standard deviation. Increasing both the heating time and electric current increases the irregularity of the temperature distribution. This is particularly evident for a heating time of 4 s. In this case, a high standard deviation (as high as 34.363 °C for 1000 A) is seen despite the high temperature being reached. This can also be observed in the distribution pattern (Figure 7), a sudden decrease is visible as the edge of the insert is approached. Therefore, the part of the forming surface with a uniform temperature distribution is reduced. Especially in the case of the selection of parameters which slightly exceed 200 °C (e.g., 2 s 1000 A), this decrease is visible, which means that the desired temperature is not reached on the entire forming surface, but it is limited on its part (for this case 3.9 mm) from the edge. This may be reflected in the properties of the manufactured object, inhomogeneity of properties between its center and edges.

### 3.3. Selection of Process Parameters

**Figure 8:**
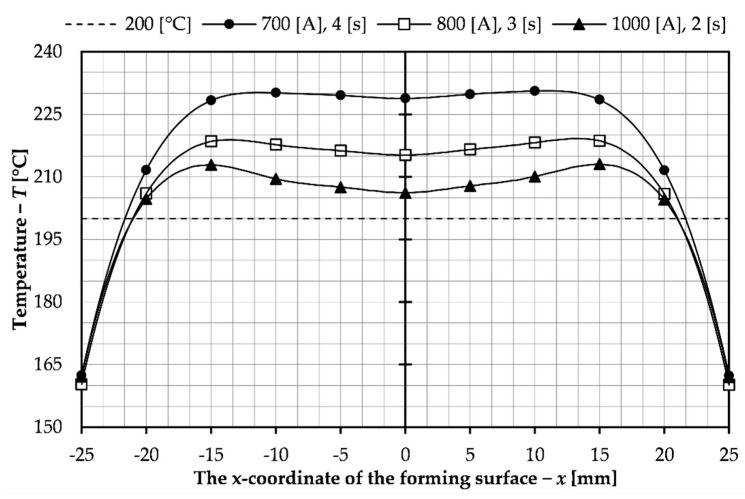
Temperature distribution on the surface of the forming insert for selected sets of parameters.

Based on the data presented, the sets of parameters used to control the process, that is, heating time and electrical current, were selected to achieve a temperature of 200 °C on the forming surface of the slider for the presented sliders system with a detachable inductor. Three sets were selected from a series of previously conducted tests. The results of the temperature distribution on the forming surface for the tests conducted with these parameters are collected in Figure 8.

**Figure 9:**
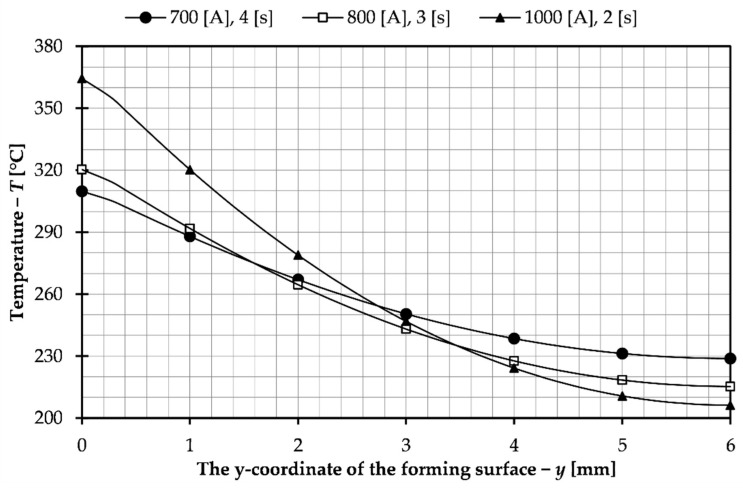
Temperature in the depth of the forming insert for selected sets of parameters.

Moreover, the temperature distribution inside the insert was investigated, as shown in Figure 9. The purpose of this tests was to check if the insert material would not overheat and, consequently, the material properties of the insert would be damaged.

The parameters chosen were those for which a temperature of 200 °C was reached, but not along the entire length of the forming surface. This is due to the analysis of the results presented in Table 2. Sets that allow this temperature to be reached along the entire length of the forming surface reach too high maximum temperatures as well as inside the insert, exceeding 400 °C, which could cause overheating and damage of the tool. For none of the selected parameters this value was exceeded, which can be seen in the graph in Figure 9. In Table 3, which compares the parameters for the selected sets, the length of the insert surface on which the temperature of 200 °C was achieved from the described aspects was also included. As can be seen, the values are similar for all sets, but the best results were obtained for time 4 s and intensity 100 A. From the economical point of view, the most beneficial solution is to use high electric current 1000 A and short insert heating time 2 s. It would allow obtaining a faster production process for the injection mold. However, in this case, there is less even heating of the insert and much higher temperature on the coil side, which can lead to faster wear of the tool. A much more uniform temperature distribution occurs for 700 A and a heating time of 4 s. A higher forming surface temperature was also obtained. The disadvantage of these parameters is the significantly increased process time which has an impact on production costs. Moreover, a greater difference in temperature as can be seen in the data shown in Table 3 and in the graphs presented despite achieving the greatest length with the desired temperature. For these reasons the best solution would be to select intermediate parameters, an example of which is the third set selected from the performed simulation tests. In this case the current was 800 A and the heating time 3 s. As can be seen from the graphs and presented data, these parameters allowed for a smaller variation of temperature in the insert and the time was not significantly increased. The selected sets of process parameters are to allow analysis of their influence on the induction heating of the insert of the injection mold with sliders and to choose the best parameters for possible application and experimental tests.

## 4. Discussion

This paper presents an investigation and a description of the concept of a detachable inductor integrated with the sliders. Such a solution would allow the application of the RTC technology, which is selective induction heating of molding surfaces in the injection mold with sliders. As a result, it would be possible to obtain a good filling of the cavity, especially for parts with more complex shapes. It would also positively influence the mechanical properties of the molded part as well as the quality of the product and repeatability of production. Several simulation studies of induction heating relevant to the injection molding process have been conducted in this paper. The influence of the device geometry and the influence of the magnetic concentrator on the obtained results were studied. The analysis of these results allowed us to carry out further series of tests in which the influence of process parameters such as frequency of the current flowing through the inductor, electric current, and heating time on the obtained results was examined.

As a result of the research the influence of the magnetic concentrator on the heating process was analyzed, its beneficial influence on the obtained results can be clearly stated. Application of the concentrator allowed to obtain more than twice higher temperatures of the forming surface for the same process parameters. This is due to the reduction of the heating of the slider body, which also translates into a higher efficiency of the process. The effect of the shape of the concentrator itself on the temperature distribution can also be seen in the results. By changing the shape, the heating of the slider body was reduced and more favorable temperature distribution on the forming surface was achieved. At the same time, an undesirable phenomenon, i.e., the formation of areas with increased temperature, was eliminated. Choosing the most favorable solution allowed to analyze the influence of process parameters on the results. The first one was the frequency of the electric current. The value of 10 kHz was chosen from the range selected for the study due to the in-deep character of induction heating in the presented design.

The next part of the study examined the effect of heating time and electric current. In order to achieve the desired temperature and favorable temperature distribution on the surface, it is necessary to choose both parameters while keeping the previous properties constant. The tests showed that it was not possible to achieve the desired results for a time of 1 s. In the case of the described solution, it was assumed that the process time cannot exceed 4 s in order not to increase the total process time. Such a limitation is also advantageous for the process because increasing the time causes a significant increase in the inequality of the temperature distribution, which can be seen in the presented results. Additionally, increasing the electric current causes an increase in the irregularity of the temperature distribution. For the parameters chosen in the study, this is evident for the high values of both parameters. For the extreme values, that is, 4 s and 1000 A, a standard deviation as high as 34.363 °C is achieved. This is due to the significant decrease in temperature approaching the edge of the insert which is associated with the lower intensity of the magnetic field in these areas and heat conduction. This resulted in the inability to reach temperatures above 200 °C over the entire surface of the insert without overheating the core. For this reason, the final parameter sets selected for the broader analysis do not reach this temperature over the entire length, but only in the greater part of it. For the selected parameters, these lengths range from 42.2 to 43.6 mm (per 50 mm insert height). This results in a limitation of the molding area of the insert itself. Depending on the part to be molded, this would entail adjusting the height of the insert to achieve the desired temperature on the molding part for the chosen process parameters. The irregularity that can be observed would be associated with a change in both the strength and aesthetic properties of the product. Especially when the temperature decrease is with a steeper slope.

The simulation studies presented in this paper have allowed the selection of parameter sets that would make it possible to carry out a series of experimental studies to validate the results obtained and to select the optimum operating parameters of such a device. The presented shape of the molding in the case studied is uncomplicated, but at the same time technological. The device is not only limited to the selected product dimensions, depending on the product, the construction of the detachable inductor together with the whole mold could be customized. Analysis of a more complex shape of the molded part, and with different dimensions, could allow obtaining more diverse results. This would also allow for a better opportunity for magnetic concentrator shape studies on the results for such a part produced. An important limitation in the presented case is the possibility of heating only with a closed mold, which should be considered in the case of building a prototype.

The conducted analysis and studies have allowed to describe the solution of induction heating in the injection mold with sliders. The purpose of applying such technology is to achieve better filling of the molding cavity and to obtain better product properties. This is confirmed by the results of the research in which the influence of increasing the mold temperature was studied. It was possible to obtain better strength properties [36,37,38], an example of this is the increase in strength of flexible hinges [39]. As well as aesthetic properties of the products [29,40,41]. It is also important to increase the repeatability of the process and possibly eliminate additional manufacturing processes. Due to the visible trend in the use of waste, especially biological [42,43], the authors also do not exclude the use of this technology in the injection molding of plastics with different types of fillers to improve the quality of the product and the process itself.

Through research and analysis, it was possible to confirm the validity of the presented concept. This allows further work on the solution of induction heating for injection molds. The conducted research allowed the selection of a number of parameters, which can be the basis for the experimental studies. The analysis of the concentrator’s influence allows evaluating its influence on the process, especially its shape, which should be taken into account in the design process. As a result of this research, it is possible to further work on increasing the quality of parts produced by injection molding and improving the process itself.

## Figures and Tables

**Table 1:** Material parameters used in the experiments.

Material	*μ* [-]	$\sigma \;\left[\frac{S}{m}\right]$	$\lambda \;\left[\frac{W}{K\cdot m}\right]$	$C\;\left[\frac{J}{kg\cdot K}\right]$	$\rho \;\left[\frac{kg}{m^{3}}\right]$
Steel 1.2343	60	107	45	460	7800
Copper	1	5.6 × 10^7^	370	385	8700
Fluxtrol A	130	5 × 10^−5^	23	430	6600
Air	1	0	0.025	1005	1

**Table 2:** Summary of average, median and standard deviation values for simulation tests performed for different heating times and different electric currents.

Time *t* [s]	Electric Current *I* [A]	Average Value [°C]	Median Value [°C]	Standard Deviation [°C]
1	500	39.416	39.499	0.858
	600	47.960	48.079	1.235
	700	58.056	58.218	1.681
	800	69.706	69.918	2.196
	900	82.909	83.177	2.779
	1000	97.666	97.996	3.431
2	500	66.226	66.247	2.660
	600	86.565	87.718	3.830
	700	110.602	112.172	5.213
	800	138.338	140.388	6.808
	900	169.771	172.366	8.617
	1000	204.903	208.107	10.638
3	500	94.367	96.638	5.304
	600	127.089	130.359	7.637
	700	165.760	170.211	10.395
	800	210.381	216.194	13.577
	900	260.950	268.308	17.184
	1000	317.470	326.553	21.214
4	500	122.134	126.629	8.591
	600	167.074	173.546	12.371
	700	220.184	228.993	16.838
	800	281.464	292.971	21.992
	900	350.916	365.479	27.834
	1000	428.538	446.517	34.363

**Table 3:** Summary of average, median and standard deviation values for simulation tests performed for selected sets of parameters.

Time *t* [s]	Electric Current *I* [A]	Average Value [°C]	Standard Deviation [°C]	Distance with the Desired Temperature [mm]
2	1000	204.903	10.638	42.2
3	800	210.381	13.577	42.4
4	700	220.184	16.838	43.6

## Data Availability

The data presented in this study are available on request from the corresponding author.
